# An automated pipeline for analyzing medication event reports in clinical settings

**DOI:** 10.1186/s12911-018-0687-6

**Published:** 2018-12-07

**Authors:** Sicheng Zhou, Hong Kang, Bin Yao, Yang Gong

**Affiliations:** School of Biomedical Informatics, University of Texas Health Science Center at Houston, 7000 Fannin Street, Suite 600, Houston, 77030 TX USA

**Keywords:** Medication events, Patient safety, Event reporting, Machine learning

## Abstract

**Background:**

Medication events in clinical settings are significant threats to patient safety. Analyzing and learning from the medication event reports is an important way to prevent the recurrence of these events. Currently, the analysis of medication event reports is ineffective and requires heavy workloads for clinicians. An automated pipeline is proposed to help clinicians deal with the accumulated reports, extract valuable information and generate feedback from the reports. Thus, the strategy of medication event prevention can be further developed based on the lessons learned.

**Methods:**

In order to build the automated pipeline, four classic machine learning classifiers (i.e., support vector machine, Naïve Bayes, random forest, and multi-layer perceptron) were compared to identify the event originating stages, event types, and event causes from the medication event reports. The precision, recall and F-1 measure were calculated to assess the performance of the classifiers. Further, a strategy to measure the similarity of medication event reports in our pipeline was established and evaluated by human subjects through a questionnaire.

**Results:**

We developed three classifiers to identify the medication event originating stages, event types and causes, respectively. For the event originating stages, a support vector machine classifier obtains the best performance with an F-1 measure of 0.792. For the event types, a support vector machine classifier exhibits the best performance with an F-1 measure of 0.758. And for the event causes, a random forest classifier reaches an F-1 measure of 0.925. The questionnaire results show that the similarity measurement is consistent with the domain experts in the task of identifying similar reports.

**Conclusion:**

We developed and evaluated an automated pipeline that could identify three attributes from the medication event reports and calculate the similarity scores between the reports based on the attributes. The pipeline is expected to improve the efficiency of analyzing the medication event reports and to learn from the reports in a timely manner.

**Electronic supplementary material:**

The online version of this article (10.1186/s12911-018-0687-6) contains supplementary material, which is available to authorized users.

## Background

Preventing medication events is a major priority for the United States health system [1, 2]. The rate of medication events in hospitals is reported between 4.8 and 5.3% [1, 3, 4]. The events may cause substantial adverse consequences to patients, including but not limited to the patient harms, unnecessary hospital admissions, additional resource utilization, and delay of daily work [5, 6]. According to the Institute of Medicine (IOM)’s report -- *To Err is Human*, about 7000 deaths each year are related to medication events [7]. Moreover, it is estimated that medication events cause 1 of 131 outpatient and 1 of 854 inpatient deaths in hospitals [7]. In the view of the prevalence of medication events and the resultant adverse consequences, improving medication safety has become a global priority [8].

Medication event reporting is a significant way for reducing medication errors and developing error prevention strategies [7]. Hospitals and federal agencies in the US have established their own event reporting programs to manage the medication event reports. However, the event reporting systems are overly focused on collecting reports rather than helping healthcare providers learn from the events [9, 10] and analyze the reports to enhance medication safety [11]. The prevalence of reporting systems results in an exponential amount of event reports, which impedes real time analysis of event reports [11]. Thus, an automated mechanism is in an urgent need to facilitate the analysis and management of collected event reports.

Data mining methods are adopted extensively in analyzing the patient safety event reports [12]. Advanced computational methods, such as nature language processing (NLP), statistical analytics, and machine learning algorithms, could transform biomedical data into meaningful knowledge and improve patient safety [13]. Prior studies applying data mining methods to extract the medication events from the biomedical literature, social media and medication event reports [14–19] have validated the feasibility and efficiency of data mining methods in dealing with medication events. To identify the patient safety event reports, researchers have applied machine learning methods [20–23] for unveiling the event reports under miscellaneous category and classifying the reports into sub-groups. The studies on the general patient safety event reports paved a path for developing automated pipelines applicable for analyzing medication event reports.

Beyond the technique perspective, it is essential to consider the nature of medication events and event report analysis workflow when designing an automated analysis tool. The challenge resides in the categorization of medication events for learning. Our preliminary work demonstrated the importance of the medication error originating stages in clinical settings by applying data mining methods to identify the stages [12]. Besides the event originating stages, event type and cause are further included for understanding the events and developing event prevention strategies [2, 11]. In this study, we designed and developed a two-step pipeline that can identify three attributes of events, i.e. event originating stage, event type and event cause from the medication event reports; and re-organized the similar event reports based on these three attributes. Medication events are often complicated because they spread across multiple stages ranging from medication ordering to monitoring and reconciliation process in the healthcare settings, and the event types and causes can be obscured by ambiguity and incompleteness of event reports. To clarify how an event happens from the origination as well as its type and cause, several tools can be relied on. The partitions of the medication error originating stages are highly consistent among the guidelines developed by authoritative agencies, e.g. The Food and Drug Administration (FDA), World Health Organization (WHO), The Agency for Healthcare Research and Quality (AHRQ) and The National Coordinating Council for Medication Error Reporting and Prevention (NCC MERP) [24–27]. Event types and event causes in the reports can be classified based on the NCC MERP Taxonomy of Medication Errors, a well-recognized taxonomy designed for recording, tracking, categorizing and analyzing the medication events, with standard language and structure for medication error related data [28, 29]. With the help of these tools, identification and categorization of event originating stage, type and cause of medication events can provide an overview of a medication event report, which would simplify the manual review process and benefit clinicians learning from the events.

Based on the identified attributes, we further proposed a similarity measurement to facilitate re-organizing the reports. The similarity measurement is a fundamental problem widely applied in bioinformatics, computational linguistics and NLP [30]. Recently, measuring similarity has become one of the mainstream topics in clinical informatics research, since it could organize clinical or patient data into groups and help researchers better understand the characteristics of each group [31]. Approaches to measure the semantic similarity are categorized as edge-based [32] and node-based approaches [33], or as pairwise [31, 34] and groupwise approaches [35, 36]. We employed the groupwise approach to develop the similarity measurement, taking its advantage in comparing the term sets from a macro view instead of integrating similarity between individual terms [37]. We then evaluated the feasibility of our proposed pipeline using both machine learning evaluation metrics and human subject evaluation. Compared to the traditional manual review approach, our pipeline is expected to reduce the workload of patient safety experts in analyzing the event reports and identifying valuable information from the reports for the purpose of shared learning.

## Methods

### System overview

**Fig. 1:**
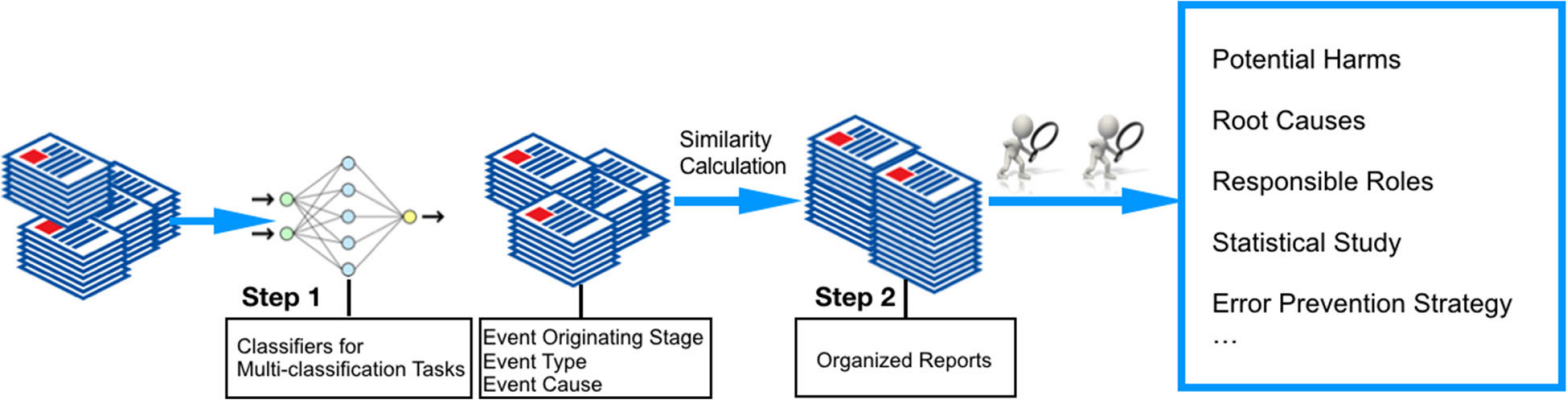
An overall sketch of the proposed automated pipeline for analyzing medication event reports

To build the automated pipeline, we need to complete three multi-classification tasks. Each report was classified by three attributes, i.e. event originating stage, event types and event causes. The three attributes of a report were labelled to construct a vector that represents the report. The three-dimensional vectors would be later applied to calculate the similarity between reports according to our proposed measurement. We applied classic machine learning metrics, including precision, recall, and F-measure to evaluate the performance of multi-classification tasks. We also developed a questionnaire for domain experts to evaluate the similarity measurement. Figure 1 shows the workflow of our automated pipeline.

### Data preparation

The medication event reports in the AHRQ common formats were submitted by hospitals to a Patient Safety Organization (PSO) in 2016 [38]. Each report contains both structured data and unstructured narratives. The narratives describe the detailed information of the event beyond the structured data. Two patient safety domain experts with pharmacy or clinical background annotated the reports. The annotation criteria include: 1) A cutting line (fewer than 10 words) was used to exclude the reports without adequate information for the classification task. 2) The reports that describe irrelevant events were removed, i.e., the reports not mentioning any medication or describing other types of errors (e.g., device errors). 3) Each of the remaining reports was annotated in three attributes, i.e. event originating stage, event type and event cause. Labels in the three attributes are summarized in Table 1.

**Table 1:** Labels in event originating stage, event type and event cause

Attributes	Labels per NCC MERP Taxonomy
Event Originating Stage	“ordering”, “transcribing”, “dispensing”, “administering”, “monitoring”, “medication reconciliation”
Event Type	“wrong dose”, “wrong dose (omission)”, “wrong drug”, “wrong time”, “wrong record”, “billing issue”, “adverse drug reaction” and “wrong administration”
Event Cause	“information deficit”, “performance deficit”, “devices (HIT)”, “pathophysiological factor”, and “external factor”

All the labels were extracted and adapted from the medication error taxonomy developed by NCC MERP [27]. Due to the constraint of the report quality, reports not containing the cause of event were labeled with “external factor”. Two experts reviewed the reports and any divergence on the annotations was resolved through group discussion.

### Feature extraction

To implement the multi-classification tasks, we applied a validated NLP workflow to pre-process the medication event reports [12]. All the numbers and punctuations in the reports were removed, and the words in a plural form were converted to a singular form. All words were transformed to lower cases. The tenses of the sentences were unified to simple present tense. The Snowball stemmer was applied to transform the terms to their root forms [39]. Rainbow stop word list was applied to remove the stop words [40]. After pre-processing, the features were extracted from the texts. The goal of feature extraction is to transform the text data into numerical representations that are interpretable by classifiers while providing discriminative information for classification [20]. To extract features, N-grams tokenizer was used to split a string of text into term vectors. Each vector contains one to three words. The reports were represented as a bag-of-words (BOW) model, a widely applied model in document classification to extract features [41]. In this model, the text in each report is represented as a bag of the unique words or word groups in the text. The word order and grammar are ignored in this model. Then, the term frequency-inverse document frequency (TF-IDF) was applied to transform the BOW matrix into a numeric representation [42]. The term vectors in the BOW matrix were used as features for the text classification tasks. In order to avoid the high redundant features, the high dimensionality of the feature space was reduced by the information gain algorithm, which is commonly used in text classification tasks [43]. We ranked all term vectors and chose the top 0.5% as final features since the contributions of the features below the threshold are negligible.

### Text classification

There are mainly two types of classic machine learning models, the discriminative model (e.g. support vector machines (SVM), random forest, and simple neural network) and generative model (e.g. Naïve Bayes). Generally, the generative models are typically more flexible than discriminative models in expressing dependencies in complicated learning tasks, while the discriminative classifiers outperform the generative classifiers in text classification of high-dimensionality data task with limited sample size [20, 44]. According to our preliminary work, the SVM, random forest, Naïve Bayes and multi-layer perceptron were proved effective in performing the text classification tasks when applied to similar event reports [12]. Thus, both generative and discriminative models were tested in our study to perform the text classification tasks, which includes SVM, random forest, Naïve Bayes and MLP algorithms. The grid search method was used to optimize the parameters for the algorithm implementation [45]. The ZeroR algorithm was used as baseline classifier. The benchmark comparisons were performed among these algorithms.

### Similarity measurement of medication event reports

We proposed a similarity measurement to identify and group similar medication event reports based on the results of multi-classification tasks. Three labels, error originating stage, type, and cause, were assigned to each report. The three labels compose a three-dimensional vector that represents a report. The similarity between two reports is calculated using the cosine similarity for vector space models [46].

1 $$ \mathrm{Similarity}=\cos \theta =\frac{\mathrm{A}\cdot \mathrm{B}}{\parallel \mathrm{A}\parallel \parallel \mathrm{B}\parallel }=\frac{\sum_{i=1}^n{A}_i{B}_i}{\sqrt{\sum_{i=1}^n{A}_i^2}\sqrt{\sum_{i=1}^n{B}_i^2}} $$

The **A** and **B** are the vectors, *A*_*i*_ and *B*_*i*_ are the components of the vectors.

Table 2 shows an example of the similarity measurement. Report_1 and Report_2 were both labeled with three identical labels, “Administration”, “Wrong Dose” and “Performance Deficit”. According to our measurement, the similarity score (Repor_1 v.s. Report_2) = 1, which means they are highly similar or identical. Actually, Report 1 and 2 describe two medication errors in clinical settings with common errors in nature. In brief, they both describe a medication event that happened during the administration stage, and a nurse gave patient wrong dose of drug (overdose) due to poor performance. This type of error can be preventable if the nurses check the order and scan the drug before the administration.

**Table 2:** Two similar medication event reports

Cases	Report Details
Report_1	Patient ordered: Take 1/2 of Drug A 0.5 mg tab for total dosage of 0.25 mg TID. When looking at the narc book to check what had been signed out since yesterday I noticed that [x] who gave the patient’s AM dose did not 1/2 the tablet that she gave. I double checked with the destruction log to see if anything was wasted and it was not. Patient received 0.5 mg instead of 0.25 mg. Informed adult day nurse [x] who will follow up with the charge nurse and inform the physician.
Report_2	39 units of Drug A drawn and administered instead of the required 14 units of Drug A as ordered. (Does have an order for 25 units of Drug A not QID)

### Evaluation

We used a stratified 10-fold cross validation method to evaluate the classifier performances.

To calculate the similarities between event reports, we conducted an empirical evaluation to test the feasibility of our similarity measurement. The evaluation, in the form of a questionnaire (see Additional file 1), was conducted regarding whether the results produced by our similarity measurement are consistent with the results produced by domain experts. The questionnaire was produced by domain experts and reviewed in term of face and content validities by a PSO, and then distributed and collected using the Google form, an online tool developed by Google. The University Institutional Review Board approved the questionnaire. An eligible participant of the study should be a nurse with at least one time reporting experience on medication events in clinical settings. Responses were received from a PSO and university nursing schools.

The questionnaire contains ten multiple-choice questions. Each question contains a target medication event report and four optional reports in a randomized order. The four randomized optional reports imply a similarity gradient calculated by the measurement in contrast to the target report. The gradient in similarity is represented by a 4-point ordinal scale, ranging from “different” to “similar”. We chose narcotics, one type of the high-alert drugs, as a representative to minimize the impact of variation of medication names [47]. The target report and four options were chosen using stratified sampling method according to the distributions of the label combinations of the reports. The principle is to maximize the coverage of the types in the label combinations. Considering the clinical workflow, clinicians tend to study similar reports as groups to identify patterns of the medication events. Thus, participants were asked to select the most similar report in the options to a target report. The accuracies were measured as evaluation metrics to test whether the pre-calculated gradient is in accordance with decisions of human experts.

Table 3 shows an example question of the questionnaire. According to our similarity measurement, the similarity scores between the **Target Report** and **Reports A, B, C** and **D** are [0.667, 0, 0.333, 1]. Two standards, a strict standard and a loose standard were applied to interpret the answers. For the strict standard, participants are expected to select the **Report D**, which has a similarity score of 1 with the **Target Report**, as the correct answer. According to our measurement, they are “identical” reports. As shown in Table 2, the **Target Report** and **Report** **D** describe two clinically similar medication events in hospitals. The two events all happened during the medication administration stage that the nurses gave the medications at wrong time. For the loose standard, either **Report** **A** or **D** can be considered correct. Report **A**, which has common attributes to the **Target Report**, describes that a nurse gave the medication to the patient at wrong time. Nevertheless, that was due to the order time was wrong and the manual order was not merged. The event originated in the medication ordering stage instead of the administration stage.

**Table 3:** An example of multiple-choice questions in the questionnaire

Reports	Report Details
Target Report	Patient given 60 mg Drug A ivp 4.5 h early than scheduled time. Dr. [x] called and said hold Drug B for 2 hours. Pt showing no signs or symptoms of reaction to early dose.
A	Patient was ordered Drug A 0.1 mg PO QHS. The order was put in with the correct directions and wrong time. [x] gave the patient Drug A 0.1 mg at 06:30 instead of 21:30 on 9/16/16. Pharmacy did not merge the manual order yesterday with their order so the patient also received 0.1 mg at 21:30 on 9/15/16. I discontinued the manual order and informed [x].
B	Patient given Drug A and developed redness and rash, drug discontinued, given Drug B.
C	Pyxis drawer failed and never opened when trying to remove 4 5 mg Drug A. Drawer then recovered with [x]. oxy count was then off, report showed that I had pulled the meds which I had not. [x] was also a witness.
D	I went into the room at 16:30, to give the patient her 17:00 meds. While in the room, I asked the patient if she was in pain. She stated she was and would like a pain pill. Without double checking the MAR I pulled the patients Drug A and gave it to her. When I informed the nurse that I had given her the drug, she stated the next dose is scheduled at 20:00.

## Results

**Fig. 2:**
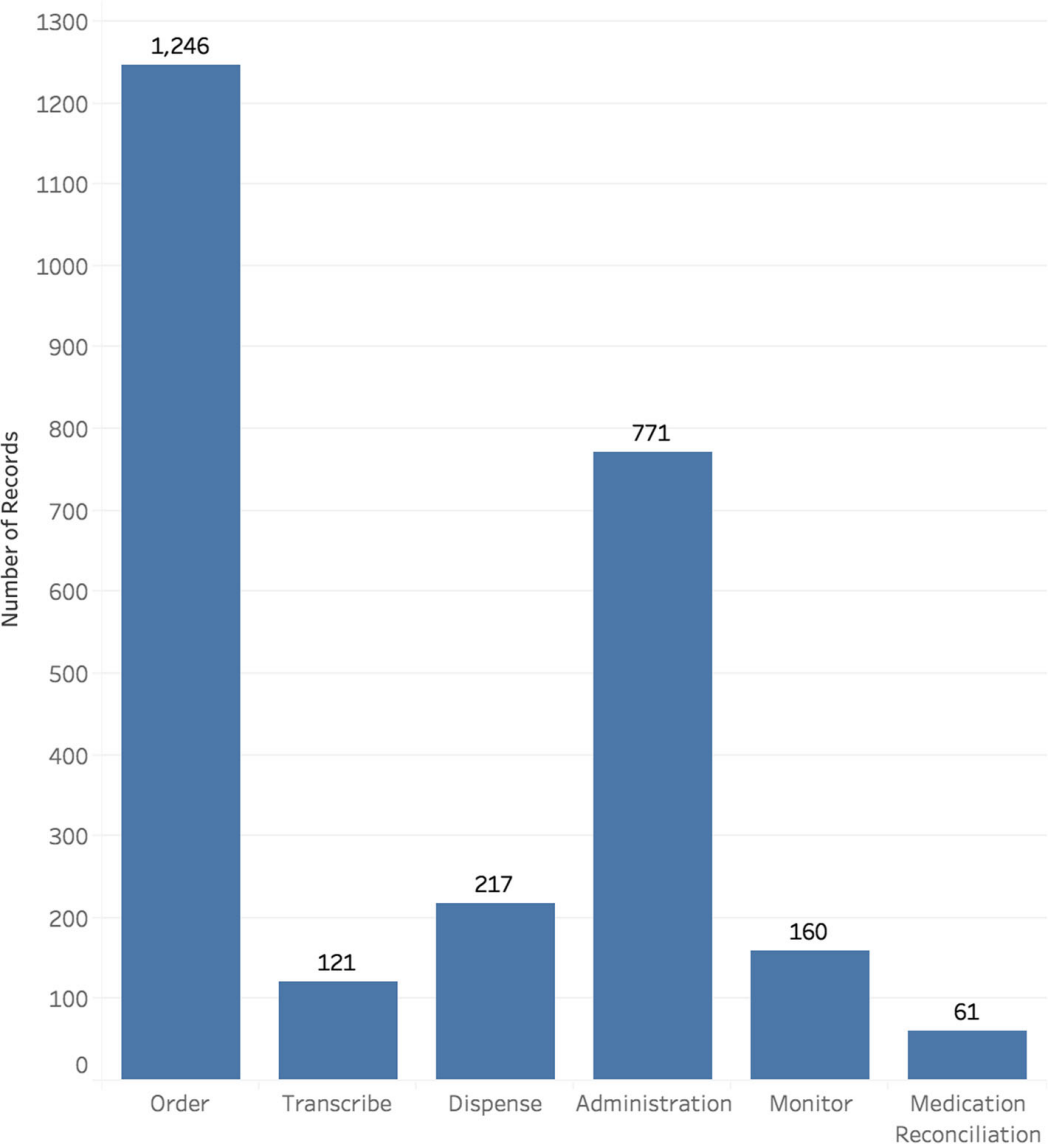
Distributions of the annotated event originating stages of the medication event reports

**Fig. 3:**
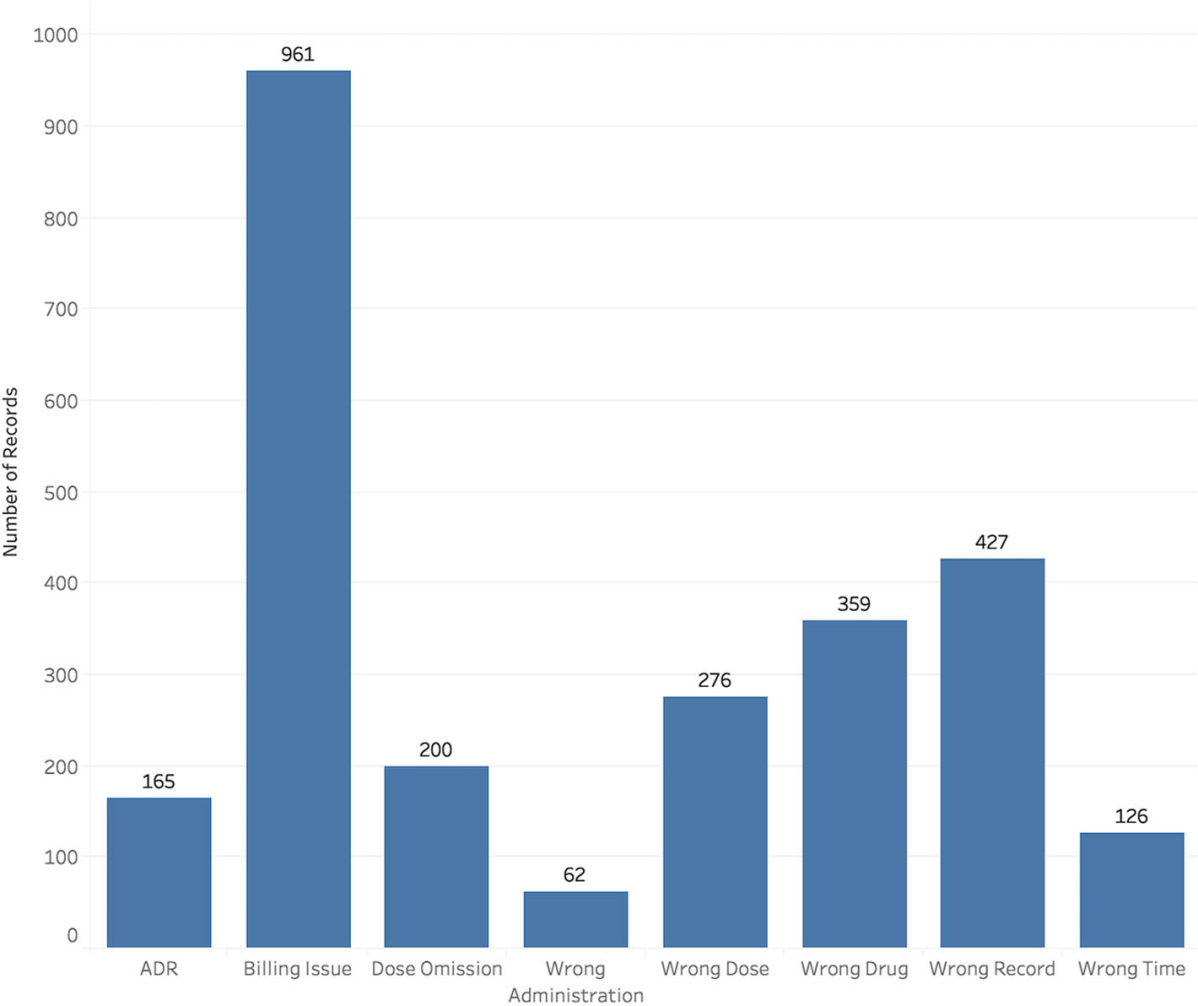
Distributions of the annotated event types of the medication event reports

**Fig. 4:**
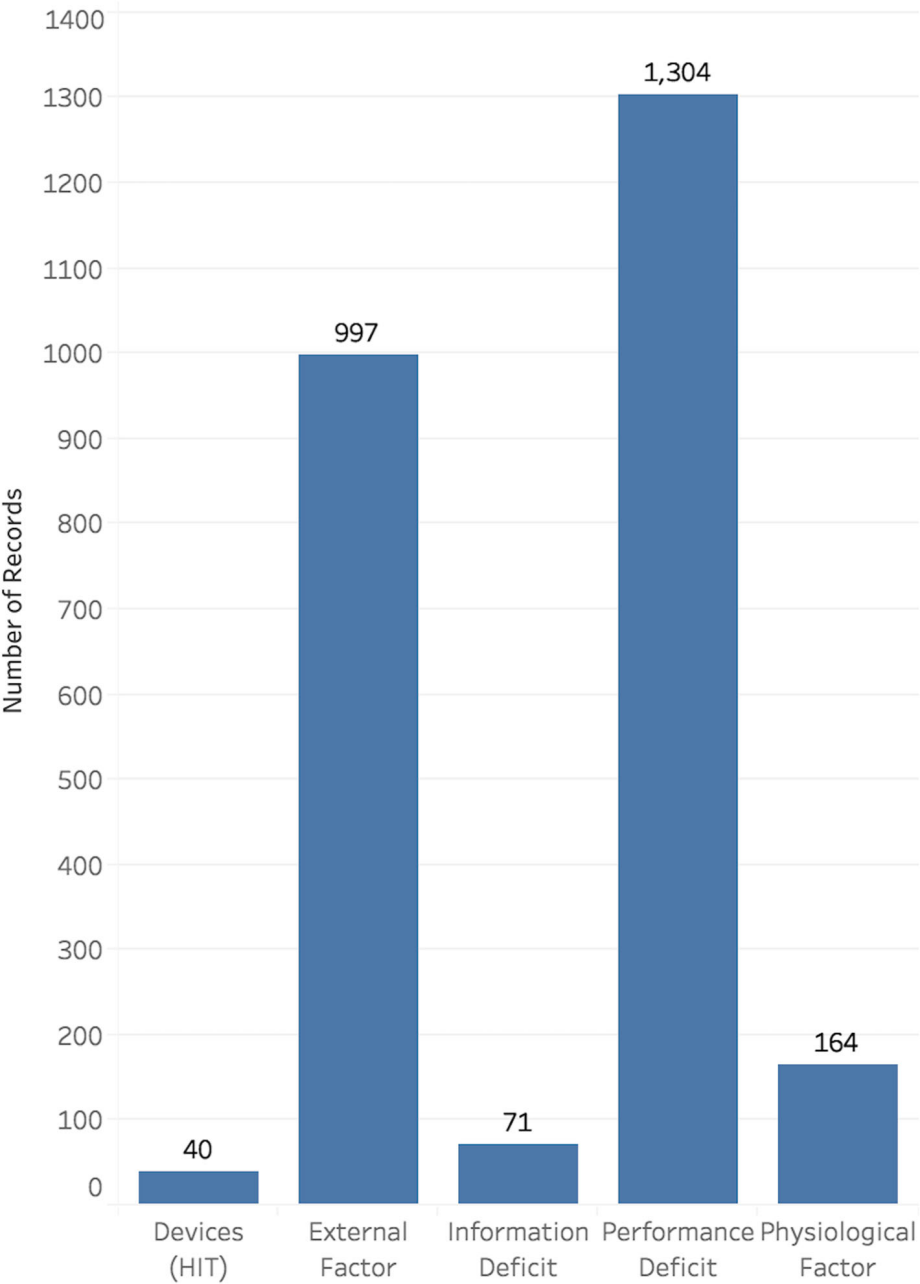
Distributions of the annotated event causes of the medication event reports

According to the annotation criteria, a total of 2576 medication event reports were included in the study. The distributions of the data annotation results are shown in Figs. 2, 3, and 4.

The distributions of the annotated labels of reports under three attributes are not balanced. As shown in Fig. 2, the events happened most frequently during the ordering and administration stages. For the medication event types, the most frequent one is ‘billing issue’, a special type of medication events in hospitals related to the health information technology (HIT) and administration system in hospitals. For the event causes, the “performance deficit” of clinicians occupies more than 50%. The reports with label of “External factor” occupy about 38%, but these event reports contain little information about the event causes. Basically, different error types have various error originating stages and causes, except the ‘billing issue’, which only happened in the ordering stage, and the ‘adverse drug reaction’ errors, which were only caused by pathophysiological factor.

### Identifying the event originating stages, event types and event causes

A BOW matrix with 79,821 vectors was obtained, and 399 (0.5%) of them were kept as final features for the multi-classification tasks according to the information gain algorithm. We tested the SVM, Random Forest, Naïve Bayes and Multi-layer perceptron algorithms to accomplish the tasks of identifying the event originating stages, event types and causes. The parameters of the classifiers were optimized by grid search method.

The performances of the baseline classifier (ZeroR) are shown in Table 4. Tables 5, 6 and 7 show the best performances of the classifiers for identifying the event originating stage, event type and cause. SVM classifiers exhibit the best performance for identifying the event originating stages and event types. A random forest classifier achieves the best performance for identifying the event cause.

**Table 4:** Performances of ZeroR classifier for identifying the error originating stages, types and causes

Classification Task	Overall Precision	Overall Recall	Overall F-Measure
Event Originating Stage	0.234	0.484	0.315
Event Type	0.139	0.373	0.203
Event Cause	0.256	0.506	0.340

**Table 5:** SVM implementation for identifying the event originating stages

Event Originating Stage	Precision	Recall	F-Measure
Ordering	0.895	0.892	0.894
Transcribing	0.464	0.430	0.446
Dispensing	0.612	0.502	0.552
Administering	0.735	0.797	0.765
Monitoring	0.768	0.730	0.748
Medication Reconciliation	0.778	0.700	0.737
Overall	0.792	0.795	0.792

**Table 6:** SVM implementation for identifying the event types

Eevnt Type	Precision	Recall	F-Measure
Adverse Drug Reaction	0.766	0.873	0.816
Billing Issue	0.978	0.978	0.978
Wrong Dose	0.493	0.540	0.516
Wrong Dose (Omission)	0.640	0.550	0.591
Wrong Record	0.871	0.857	0.864
Wrong Drug	0.497	0.682	0.575
Wrong Time	0.621	0.143	0.232
Wrong Administration	0.727	0.129	0.219
Overall	0.778	0.769	0.758

**Table 7:** Random forest implementation for identifying the event causes

Event Cause	Precision	Recall	F-Measure
Performance Deficit	0.856	0.978	0.913
Information Deficit	0.714	0.070	0.128
Devices (HIT)	0.632	0.126	0.210
Pathophysiological Factor	0.896	0.628	0.738
External Factor	0.979	0.947	0.963
Overall	0.927	0.927	0.925

### Human subject evaluation for the similarity measurement of medication event reports

We received 11 responses to our evaluation questionnaire. All the participants are registered nurses, who are experienced in reporting medication events in clinical settings.

Two standards were applied to determine the accuracies of the collected answers. For the strict standard, the average accuracy for the questionnaires is 80.9%, and for the loose standard, the average accuracy is 93.6%. Under the strict standard, the highest accuracy of a single question is 91.0%, while the lowest accuracy is 54.5%. For the loose standard, the highest accuracy of a single question is 100%, while the lowest accuracy is 81.8%.

Table 8 shows the accuracies of the 11 participants’ answers under the two standards. One participant only obtained 20% accuracy under the strict standard and 50% accuracy under the loose standard. We estimate this participant did not correctly understand our questionnaire.

**Table 8:** The two-standard accuracies of the answers from the 11 participants

Participant ID	1	2	3	4	5	6	7	8	9	10	11
Accuracy (strict standard)	90%	70%	90%	20%	80%	100%	100%	90%	100%	60%	90%
Accuracy (loose standard)	100%	90%	100%	50%	100%	100%	100%	100%	100%	90%	100%

## Discussions

### Main findings and implications

Valuable information in medication event reports indicates how and why the medication events happened in clinical settings, which are deemed helpful in identifying the root casues and prevention strategies in medication safety. Our work was inspired by the workflow of analyzing medication event reports in clinical settings. The event reports are manually reviewed in a case by case manner at regular time intervals, which are inefficient and labor intensive. In addition, the collected reports are not well organized, which is a basic challenge for clinicians to review effectively and efficiently. Our proposed automated pipeline meets such an information need for improvement. The pipeline contains two steps. The first step is to identify three core attributes of a medication event from the narrative event report, the event originating stage, event type, and event cause, which are significant for summarizing the medication events in clinical settings. The F-measures for identifying the three attributes are 0.792, 0.758 and 0.925, respectively. For identifying the event types and causes, there are no benchmarks for comparisons. Thus, we applied a standard baseline classifier (ZeroR) as benchmark, the performances of our classifiers are much better than the baseline algorithm. The overall results are solid to support the second step which is to group similar reports for further manual review and study. A human evaluation was conducted to test our similarity measurement, and according our two standards, the accuracies could reach 80% and 93% respectively. The evaluation proved that our method could group the relatively similar event reports together. Analyzing the similar medication event reports in group is more likely to identify the error patterns in clinical settings and better develop the strategies for event prevention. To our knowledge, this is the very first study on the similarity among medication event reports.

Our similarity measurement is based on the medication event taxonomy, which differentiates from other works that are mainly based on the features of the texts. However, the natures of medication event reports may make them inappropriate for the traditional similarity algorithms. For example, the length of the medication event reports varies a lot, some of the reports could be more than 100 words while many of them only contain about 10 words. However, reports with 100 words and 10 words could be similar since they may describe the same medication events in clinical settings. Once our similarity measurement is integrated with the medication event taxonomy, it can be scalable and improved along with the taxonomy. For example, the NCC MERP taxonomy does not fully covere the event causes, which was reflected during our data annotation process. Some of the reports were annotated vaguely due to lack of the definition. Also, the involved personnel and medications in the medication events that are extremely important in medication events are not well defined in current taxonomies. Our similarity measurement is expected to be improved when these two attributes integrate. The proposed pipeline could be generalized to other types of patient safety events, for example, patient fall and hospital infection. The core idea is to extract the key attributes of these events based on their taxonomies, and group the similar reports based on these attributes. Also, we provided a method to evaluate the similarity measurement by designing a questionnaire that targeted to the domain experts. The questions in the questionnaire were designed to cover different levels of similarities among the reports. The results indicate that our similarity measurement is highly consistent with domain experts’ perceptions about whether two reports are similar.

### Limitations of the study

One major limitation of the study is the quantity and quality of medication event reports. The one-year PSO data may not represent the entire PSO dataset.

The distributions of the labels in the three attributes are not well balanced. For example, the reports with the labels of “ordering” and “administrating” occupy about 78% of all the reports, and the reports with other four labels in the event originating stage only occupy about 22% of the total reports. Similarly, the reports with the labels of “external factors” and “performance deficit” in the event cause occupy about 90% of all reports. The imbalanced distributions of the data resulted in low performance of our classifiers during the multi-classification tasks. A balanced distribution may help improve the performance of some sub-categories, such as “dispensing” and “transcribing” in event originating stages, “wrong time” and “wrong administration” in error types, “information deficit” and “devices (HIT)” in error causes.

The narratives of the reports vary, which requires additional steps to unify the abbreviations and variations. For instance, ‘medication’, is written as ‘med’, ‘meds’, ‘medication’, ‘drug’, ‘chemical’, ‘medicine’, etc. Those words play very similar semantics roles in the reports but will produce more word vectors than general words. More effective ways to pre-process the texts in the reports is needed. It is essential to establish a standardized reporting mechanism for reporting and identifying key attributes of the events.

The 11 participants in the evaluation show consistent results with the similarity measurement. More participants would enhance the generalizability.

## Conclusion

In order to facilitate clinicians analyze and manage the collected reports, we developed and evaluated an automated pipeline that could finish two tasks: 1) identify the event originating stages, event types and event causes; 2) re-organize the reports based on their similarities. Compared to the traditional manual review, our pipeline is expected to save time and reduce the workload for clinicians to analyze the event reports, and better discover valuable information from the reports to facilitate the development of strategies for preventing medication events.

## Additional file

Additional file 1: An automated pipeline for analyzing medication event reports in clinical settings. (DOCX 1077 kb)

## Abbreviations

AHRQ: The Agency for Healthcare Research and Quality
BOW: Bag-of-words
FDA: The Food and Drug Administration
HIT: Health Information Technology
IOM: The Institue of Medicine
NCC MERP: The National Coordinating Council for Medication Error Reporting and Prevention
NLP: Nature language processing
PSO: Patient Safety Organization
SVM: Support vector machine
TF-IDF: Term frequency - inverse document frequency
WHO: World Health Organization
