# Supplementary material for: An automated pipeline for analyzing medication event reports in clinical settings
Source: BMC Med Inform Decis Mak. 2018 Dec 7;18(Suppl 5):113. doi: 10.1186/s12911-018-0687-6 (PMC6284273; doi:10.1186/s12911-018-0687-6)

**An automated pipeline for medication event reports analysis in clinical settings**

Sicheng Zhou, Hong Kang, Bin Yao, Yang Gong*

Sicheng Zhou

Institute for Health Informatics, University of Minneapolis Twin Cities

8-100 PWB, 516 Delaware Street SE, Minneapolis, MN 55455

E-mail: [zhou1281@umn.edu](mailto:zhou1281@umn.edu)

Hong Kang

University of Texas Health Science Center at Houston

7000 Fannin Street, Houston, TX, USA

E-mail: [Hong.Kang@uth.tmc.edu](mailto:Hong.Kang@uth.tmc.edu)

Bin Yao

University of Texas Health Science Center at Houston

7000 Fannin Street, Houston, TX, USA

E-mail: [Bin.Yao@uth.tmc.edu](mailto:Bin.Yao@uth.tmc.edu)

Yang Gong

University of Texas Health Science Center at Houston

7000 Fannin Street, Houston, TX, USA

E-mail: [Yang.Gong@uth.tmc.edu](mailto:Yang.Gong@uth.tmc.edu)

*Yang Gong is the corresponding author.

**Abstract**

**Background**

Medication events in clinical settings are significant threats to patient safety. Analyzing and learning from the medication event reports is an important way to prevent the recurrence of these events. Currently, the analysis of medication event reports is ineffective and requires heavy workloads for clinicians. An automated mechanism is proposed to help clinicians deal with the accumulated reports, extract valuable information and generate feedback from the reports. Thus, the strategy of medication event prevention can be further developed based on the lessons learned.

**Methods**

In order to build the automated pipeline, four classic machine learning classifiers (i.e., support vector machine, Naïve Bayes, random forest, and multi-layer perceptron) were compared to identify the event originated stages, event types, and event causes from the medication event reports. The precision, recall and F-1 measure were calculated to assess the classifiers. Further, a strategy to measure the similarity of medication event reports in our pipeline was evaluated by human subjects through a questionnaire.

**Results**

We developed three classifiers to identify the medication event originated stages, event types and causes, respectively. For event originated stages, a support vector machine classifier obtains the best performance with F-1 measure of 0.792. For event types, a support vector machine classifier got the best performance with F-1 measure of 0.758. And for event causes, a random forest classifier reaches a F-1 measure of 0.925. A human subject evaluation was conducted to test our similarity measurement, the results show that our similarity measurement is consistent with the domain experts in the task of identifying similar reports.

**Conclusion**

We developed and evaluated an automated pipeline that could identify three factors from the medication event reports and calculate the similarity scores between the reports based on these factors. The pipeline is expected to improve the efficiency of analyzing the medication event reports, and to learn from the reports in a timely manner.

**Keywords**

Medication events; Patient safety; Event reporting; Machine learning

**Background**

Preventing medication events is a major priority for the United States health system [1, 2]. The rate of medication events in hospitals is reported between 4.8% and 5.3% [1, 3, 4]. The events may cause substantial adverse consequences for patients, including but not limited to the patient harms, unnecessary hospital admissions, additional resource utilization and delay of daily work [5, 6]. According to the IOM’s report -- *To Err is Human*, about 7,000 deaths each year are related to medication events [7]. Moreover, it is estimated that medication events cause 1 of 131 outpatient and 1 of 854 inpatient deaths in hospitals [7]. In view of the prevalence of medication events and the resultant adverse consequences, improving medication safety has become a global priority [8].

Among multiple ways for preventing medication events, medication event reporting is a significant way for reducing medication errors and developing error prevention strategies [7]. Hospitals and federal agencies in the US have established their own event reporting programs to manage the medication events. However, the event reporting systems are overly focused on collecting reports rather than learning from the events that have been reported [9, 10], and timely analyzing the reports to enhance medication safety [11]. The prevalence of reporting systems results in exponential amount of event reports, which impedes real time analysis of event reports [11]. Thus, an automated mechanism is in an urgent need to facilitate the analysis and management of collected event reports.

Data mining methods are adopted extensively in analyzing the patient safety event reports [12]. Advanced computational methods, such as Nature Language Processing (NLP), statistical analytics, and machine learning algorithms, could transform biomedical data into meaningful knowledge to improve patient safety [13]. Prior studies applying data mining methods to extract the medication events from the biomedical literature, social media and medication event reports [14-19] have validated the feasibility and efficiency of data mining methods in dealing with medication events. To identify the patient safety events from patient safety event reports, researchers have applied machine learning methods [20-23] for unveiling the event reports under miscellaneous category and classifying the reports into sub-groups according to known taxonomies or criteria. These studies focusing on the general patient safety event reports pave a path for developing automated mechanisms applicable for analyzing medication event reports.

Beyond the technique perspective, it is essential for us to consider the nature of medication events and event report analysis workflow when designing an automated analysis tool. The challenge resides in the categorization of medication events for learning from the events. Our preliminary work demonstrated the importance of the medication error originating stages in clinical settings by applying data mining methods to identify the stages [12]. Besides the event originating stages, incidence type and cause are further included for understanding the events and developing event prevention strategies [2, 11]. In this study, we designed and developed a two-step pipeline that can identify three attributes of events, i.e. event originated stage, event type and event cause from the medication event reports; and re-organized the similar event reports based on these three attributes. Medication events are often complicated because many spread across multiple stages of medication distribution process in the healthcare settings, and the event types and causes are obscured by ambiguity and incompleteness of event reports. To clarify how an event happens from the origination as well as its type and cause, several tools can be relied on. The partitions of the medication error originated stages are highly consistent among the guidelines developed by authoritative agencies, e.g. Food and Drug Administration (FDA), World Health Organization (WHO), Agency for Healthcare Research and Quality (AHRQ) and The National Coordinating Council for Medication Error Reporting and Prevention (NCC MERP) [24-27]. Event types and event causes in the reports can be classified based on the NCC MERP Taxonomy of Medication Errors, a well-recognized taxonomy designed for recording, tracking, categorizing and analyzing the medication events, with standard language and structure for medication error related data [28, 29]. With the help of these tools, identification and categorization of event originated stage, type and cause of the medication events can provide an overview of a medication event report, which would simplify the manual review process and benefit clinicians learning from events.

Based on the identified attributes, we further proposed a similarity measurement to facilitate re-organizing the reports. The similarity measurement is a fundamental problem widely applied in bioinformatics, computational linguistics and NLP [30]. Recently, measuring similarity has become one of the mainstream topics in clinical informatics research, it could organize clinical or patient data into groups and help researchers better understand the characteristics of each group [31]. Approaches to measure the semantic similarity include edge-based approach [32], node-based approach [33], pairwise approach [31, 34] and groupwise approach [35, 36]. We employ the groupwise approach to develop the similarity measurement, taking its advantage in comparing the term sets from a macro view instead of relying on the integrating similarity between individual terms [37].We then evaluate the feasibility of our proposed pipeline using both machine learning evaluation metrics and human subject evaluation. Compared to the traditional manual review approach, our pipeline is expected to reduce the workload of patient safety experts in analyzing the event reports and identifying valuable information from the reports for the purpose of shared learning.

**Methods**

**System overview**

To build the automated pipeline, we need to complete three multi-classification tasks. Each report was classified in three dimensions, event originated stage, event types and e event causes. The three labels of a report construct a vector that represents the report. The three-dimensional vectors can be applied to calculate the similarity between reports according to our proposed measurement. We applied classic machine learning metrics, including precision, recall, and F-measure to evaluate the multi-classification tasks. Then, we developed a questionnaire for domain experts to evaluate our similarity measurement. Figure 1 shows the workflow of our automated pipeline.

**Data Preparation**

The medication event reports in the year of 2016 in the AHRQ common formats were submitted by hospitals to a Patient Safety Organization (PSO) [38]. Each report contains structured data and unstructured narratives. The narratives describe the detailed information of the event beyond the structured data. Two patient safety domain experts with pharmacy or clinical background annotated the reports. The annotation criteria include: 1) A cutting line (fewer than 10 words) was used to exclude the reports without adequate information for the classification task. 2) The reports that describe irrelevant events were removed, i.e., the reports not mentioning any medication or describing other types of errors (e.g., device errors). 3) Each of the remaining reports was annotated in three dimensions, i.e. event originating stage, event type and event cause. Labels in the three dimensions are summarized in Table 1.

All the labels were extracted and adapted from the medication error taxonomy developed by NCC MERP [27]. Due to the constraint of the report quality, reports not containing the cause of event were labeled with “external factor”. The two experts reviewed all the reports and any divergence on the annotations was resolved through group discussion.

**Feature Extraction**

To implement the multi-classification tasks, we applied a validated pre-process NLP workflow to the medication event reports [12]. All numbers and punctuations in the reports were removed and the words in a plural form were converted to a singular form. All words were transformed to lower cases. The tenses of the sentences were unified to simple present tense. The Snowball stemmer was applied to transform the terms to their root forms [39]. Rainbow stop word list was applied to remove the stop words [40]. After pre-processing of the texts, the features were extracted from the texts. The goal of feature extraction is to transform the text data into numerical representations that are interpretable by classifiers while providing discriminative information for classification [20]. To extract features, N-grams tokenizer was used to split a string of text into term vectors. Each vector contains one to three words. The reports were represented as a bag-of-words (BOW) model, a widely applied model in document classification to extract features [41]. In this model, the text in each report is represented as a bag of the unique words or word groups in the text. The word order and grammar are ignored in this model. Then, the term frequency-inverse document frequency (TF-IDF) was applied to transform the BOW matrix into a numeric representation [42]. The term vectors in the BOW matrix were used as features for the text classification tasks. In order to avoid the high redundant features, the high dimensionality of the feature space was reduced by the information gain algorithm, which is commonly used in text classification tasks [43]. We ranked all term vectors and chose the top 0.5% as final features since the contributions of the features below the threshold are negligible.

**Text Classification Tasks**

There are mainly two types of classic machine learning models, the discriminative model (e.g. support vector machines (SVM), random forest, and simple neural network) and generative model (e.g. Naïve Bayes). Generally, the generative models are typically more flexible than discriminative models in expressing dependencies in complicated learning tasks, while the discriminative classifiers outperform the generative classifiers in text classification of high-dimensionality data task with limited sample size [20, 44]. According to our preliminary work, the SVM, random forest, Naïve Bayes and multi-layer perceptron were proved effective in performing the text classification tasks when applied to similar event reports [12]. Thus, both generative and discriminative models were tested in our study to perform the text classification tasks, which includes SVM, random forest, Naïve Bayes and MLP algorithms. The parameters of these classifiers needed to be optimized, the grid search method was used to find the best parameters for the algorithm implementation [45]. The ZeroR algorithm was used as baseline classifier. The benchmark comparisons were performed among these algorithms.

**Similarity Measurement of Medication Event Reports**

We proposed a similarity measurement to identify and grouped similar medication event reports based on the results of multi-classification tasks. Three labels, error originated stage, type, and cause, were assigned to each report. The three labels of a report compose a three-dimensional vector that represents the report. The similarity between two report is calculated using the cosine similarity for vector space models [46].

Similarity = $\cos\theta$ = $\frac{A\cdot B}{\parallel A\parallel\parallel B\parallel}$ = $\frac{\sum_{i=1}^{n} A_{i}B_{i}}{\sqrt{\sum_{i=1}^{n} A_{i}^{2}}\sqrt{\sum_{i=1}^{n} B_{i}^{2}}}$ (1)

The **A** and **B** are the vectors, $A_{i}$ and $B_{i}$ are the components of the vectors.

Table 2 shows an example of the similarity measurement. The Report_1 and Report_2 were both labeled with three identical labels, “Administration”, “Wrong Dose” and “Performance Deficit”. According to our measurement, the similarity (Repor_1, Report_2) = 1, which means they are highly similar or fully identical based on the labels. Report 1 and 2 describe two medication errors in clinical settings with common errors in nature. In brief, they both describe a medication event that happened during the administration stage, and a nurse gave patient wrong dose of drug (overdose) due to poor performance. This type of error was preventable if the nurses check the order and scan the drug before the administration.

**Evaluation**

The aim of multi-classification tasks is to identify the event originated stage, event type and event cause for each report, thus the performances in terms of precision, recall, and F-score were measured for each task. We used a stratified 10-fold cross validation method to evaluate the classifier performances.

To calculate the similarities between event reports, we conducted an empirical evaluation to test the feasibility of our similarity measurement. The evaluation, in the form of a questionnaire [see Additional file 1], was conducted regarding whether the results produced by our similarity measurement are consistent with the results produced by domain experts. The questionnaire was produced domain experts and reviewed in term of face and content validities by a PSO, and then distributed and collected using the Google form, an online tool developed by Google. The University Institutional Review Board approved the questionnaire. An eligible participant of the study should be a nurse with at least one time reporting on medication events in clinical settings. Responses were received from a Patient Safety Organization and Nursing school of a university.

The questionnaire contains ten multiple-choice questions. Each question contains a target medication event report and four other optional reports in a randomized order. The four randomized optional reports imply a similarity gradient calculated by the measurement in contrast to the target report. The gradient in similarity is represented by a 4-point ordinal scale, ranging from “different” to “similar”. We chose narcotics, one type of the high-alert drugs, as a representative to minimize the impact of variation of medication names [47]. The target report and four options were chosen using stratified sampling method according to the distributions of the label combinations of the reports. The principle is to maximize the coverage of the types in the label combinations. Considering the clinical workflow, clinicians tend to study similar reports as groups to identify patterns of the medication events. Thus, participants were asked to select the most similar report in options to a target report. The accuracies were measured as evaluation metrics to test whether the pre-calculated gradient is in accordance with decisions of human experts.

Table 3 shows an example question of the questionnaire. According to our similarity measurement, the similarity scores between the **Target Report** and the **A, B, C** and **D** four reports are [0.667, 0, 0.333, 1]. Two standards, a strict standard and a loose standard were applied to the answers. For the strict standard, the participants are expected to select the **D** report, which has similarity score of 1 with the **Target Report**, as the correct answer. According to our measurement, they are “identical” reports. As shown in table 2, the **Target Report** and **D** report describe two clinically similar medication events in hospitals. The two events were all happened during the medication administration stage and the nurses given the medications at wrong time. For the loose standard, the participants are expected to choose either **A** or **D** report to be considered correct. Report **A** also describes an event that a nurse gave patient the medication at wrong time. Nevertheless, that was due to the order time was wrong, thus the event originated in the medication ordering stage.

**Results**

According to the annotation criteria, there are a total of 2576 medication event reports included in the study. The distributions of the data annotation results are shown in Figure 2-4.

The distributions of the annotated labels of reports under three dimensions are not balanced. As shown in Figure 2, the events happened most frequently during the ordering and administration stages. For the medication event types, the most frequent one is ‘billing issue’, a special type of medication events in hospitals related to the Health information technology (HIT) and administration system in hospitals. For the event causes, the “performance deficit” of clinicians occupies more than 50%. The reports with label of “External factor” occupies about 38%, this part of the event reports contains little information about the event causes, as a result, the reports were labeled with “external factor”. Basically, different error types have various error originated stages and causes, except the ‘billing issue’, which only happened in ordering stage, and the ‘adverse drug reaction’ error, which only caused by pathophysiological factor.

**Identifying the event originated stages, event types and event causes**

A BOW matrix with 79,821 vectors was obtained, and 399 (0.5%) of them were kept as final features for the multi-classification tasks according to the information gain algorithm. We tested the SVM, Random Forest, Naïve Bayes and Multi-layer perceptron algorithms to accomplish the tasks of identifying the event originated stages, event types and causes. The parameters of the classifiers were optimized by grid search method.

The performances of the baseline classifier (ZeroR) are shown in table 4. Table 5-7 show the best performances of the classifiers for identifying the event originated stage, event type and cause. SVM classifiers got the best performance for identifying the event originated stages and event types. A random forest classifier got the best performance for identifying the event cause.

**Human Subject Evaluation for the Medication Event Report Similarity Measurement**

We received 11 responses to our evaluation questionnaire. All the participants are registered nurses. Eight of them are associated with a PSO institute; Three are in university Nursing school. All participants are experienced in reporting medication events in clinical settings.

Two standards were applied to determine accuracy of the collected answers. For strict standard, the average accuracy for the questionnaires is 80.9% and for loose standard, the average accuracy is 93.6%. Figure 5 shows the accuracies of the 10 questions in the questionnaire. Under the strict standard, the highest accuracy of a single question is 91.0%, while the lowest accuracy is 54.5%. For the loose standard, the highest accuracy of a single question is 100%, while the lowest accuracy is 81.8%.

Table 8 shows the accuracies of the 11 participants’ answers under two standards. One participant only got 20% accuracy under strict standard and 50% accuracy under loose standard. We estimate this participant did not correctly understand our questionnaire.

**Discussions**

**Main findings and implications**

Valuable information in medication event reports indicates how and why the medication events happened in clinical settings, which are deemed helpful in identifying the root casues and prevention strategies in medication safety. Our work was inspired by the workflow of analyzing medication event reports in clinical settings. The event reports are manually reviewed in a case by case manner at regular time intervals, which are inefficient and labor intensive. In addition, the collected reports are not well organized, which is a basic challenge for clinicians to effectively and efficiently review the reports as groups. Our proposed automated mechanism meets such an information need for improvement. This study proposed and evaluated an automated pipeline that could facilitate to extract such information. The pipeline contains two steps. The first step is to identify three core factors of a medication event from the narrative medication event report, the event originated stage, event type, and event cause, which are significant for summarizing the medication events in clinical settings. The F-measures for identifying these three factors are 0.792, 0.758 and 0.925, respectively. Compared to our previous work, the best overall F-measure for identifying the event-originated stages was slightly lower this time (0.792 vs 0.800). For identifying the event types and causes, there are no benchmarks for comparisons. Thus, we applied a standard baseline classifier (ZeroR) as benchmark, the performances of our classifiers are much better than the baseline algorithm. The overall results are solid to support the second step. The second step is to group similar reports for further manual review and study. A human evaluation was conducted to test our similarity measurement, and according our two standards, the accuracies could reach 80% and 93% respectively, which are within our expectations. The evaluation proved that our method could group the relatively similar event reports together. Analyzing the similar medication event reports in group is more likely to identify the error patterns in clinical settings and better develop the strategies for event prevention. To our knowledge, this is the very first study on the similarity among medication event reports.

Our similarity measurement is based on the medication event taxonomy, which differentiates from other works that study the document similarity. Those similarity algorithms are mainly based on the features of the texts. However, the natures of medication event reports may make them inappropriate for the traditional similarity algorithms. For example, the length of the medication event reports varies a lot, some of the reports could be more than 100 words while many of them only contain about 10 words. However, reports with 100 words and 10 words could be similar since they describe same medication events in clinical settings. Once our similarity measurement is integrated with the medication event taxonomy, it is scalable and improved along with the taxonomy. For example, the NCC MERP taxonomy has not fully covered the event causes, which is reflected during our data annotation process. Some of the reports were annotated vaguely due to the deficit of the definition. Also, the involved personnel and medications in the medication events are not well defined in current taxonomy, they are very important dimensions for medication events. Our similarity measurement is expected to be improved once integrate these two dimensions. The proposed pipeline could be generalized to other types of patient safety event reports, for example, the patient fall and the hospital infection. The core idea is to extract the core factors of these events based on their taxonomies, and group the similar reports based on these factors. Also, we provided a method that evaluates the similarity measurement. The human evaluation is required for the similarity evaluation, thus we designed a questionnaire that targeted to the domain experts. Although the similarity scores can be calculated, it is actually an ordinal scale rather than interval scale. The questions in the questionnaire were carefully designed to cover different level of similarities among the reports. The results indicate that our similarity measurement is highly consistent with domain experts’ perceptions about whether two reports are similar.

**Limitations of the Study**

One major limitation of the study is the medication event reports data in terms of quantity and quality. The one-year PSO data may not represent the entire PSO dataset.

The distributions of the labels in the three dimensions are not well balanced. For example, the reports with the labels of “ordering” and “administrating” occupy about 78% of all the reports, and the reports with other four labels in the event originated stage dimension only occupy about 22%. Similarly, the reports with the labels of “external factors” and “performance deficit” in the event cause occupy about 90% of all reports. The imbalanced distributions of the data resulted in low performances of our classifiers during the multi-classification tasks. A balanced distribution may help improve the performances of some sub-categories, such as “dispensing” and “transcribing” in event originated stages, the “wrong time” and “wrong administration” in error types, “information deficit” and “devices (HIT)” in error causes.

The narratives of the reports vary, which requires additional steps to unify the abbreviations and variations. For instance, ‘medication’, is written as ‘med’, ‘meds’, ‘medication’, ‘drug’, ‘chemical’, ‘medicine’, etc. Those words play very similar semantics roles in reports but will produce more word vectors than regular words. More effective ways to preprocess the texts in reports is needed. Also, a standard reporting mechanism for medication event will be helpful for guiding reporters during reporting and identifying key factors of the events, and thus improving the data quality.

The 11 participants in the evaluation show consistent results with the similarity measurement. More participants would enhance the generalizability.

**Conclusion**

In order to facilitate clinicians analyze and manage the collected reports, we developed and evaluated an automated pipeline that could finish two tasks: 1) identify the event originated stages, event types and event causes; 2) re-organize the reports based on their similarity. Compared to the traditional manual review approach, our pipeline is expected to save time and reduce the workload for clinicians to analyze the event reports, and better discover valuable information from the reports to facilitate the development of strategies for preventing medication events.

**List of abbreviations**

NLP: Nature Language Processing

SVM: support vector machine

AHRQ: The Agency for Healthcare Research and Quality

HIT: Health Information Technology

WHO: World Health Organization

NCC MERP: The National Coordinating Council for Medication Error Reporting and Prevention

FDA: The Food and Drug Administration

BOW: Bag-of-words

TF-IDF: Term frequency - inverse document frequency

**Declarations**

**Acknowledgements**

We thank the experts for their expertise and participation in expert review.

**Ethics approval and consent to participate**

The study has received IRB exemptions from Committee for the Protection of Human Subjects at The University of Texas Health Science Center at Houston (HSC-SBMI-18-0554) and the Louisiana Tech University (HUC 18-146).

**Availability of data and materials**

The datasets used in the study belong to the Patient Safety Organization. They are not publicly available, researchers can submit reasonable request to Patient Safety Organization to obtain the data.

**Funding**

This project is supported by Agency for Healthcare Research & Quality (1R01HS022895). Publication of this article is sponsored by Agency for Healthcare Research & Quality (1R01HS022895).

**Competing interests**

The authors declare that they have no competing interest.

**Consent for publication**

Not applicable.

**Authors' contributions**

SZ, HK and YG designed the experiments. SZ and BY prepared the data. SZ conducted the experiments and drafted the manuscript. YG and HK organized the evaluation and revised the manuscript. All authors read and approved the final manuscript.

**References**

1. Wittich CM, Burkle CM, Lanier WL, editors. Medication errors: an overview for clinicians. Mayo Clinic Proceedings; 2014: Elsevier.

2. Morimoto T, Gandhi TK, Seger AC, Hsieh TC, Bates DW. Adverse drug events and medication errors: detection and classification methods. Qual Saf Health Care. 2004;13(4):306-14.

3. Belén Jiménez Muñoz A, Muiño Miguez A, Paz Rodriguez Pérez M, Dolores Vigil Escribano M, Esther Durán Garcia M, Sanjurjo Saez M. Medication error prevalence. International journal of health care quality assurance. 2010;23(3):328-38.

4. Bates DW, Boyle DL, Vander Vliet MB, Schneider J, Leape L. Relationship between medication errors and adverse drug events. Journal of general internal medicine. 1995;10(4):199-205.

5. Bates DW, Spell N, Cullen DJ, Burdick E, Laird N, Petersen LA, et al. The costs of adverse drug events in hospitalized patients. Jama. 1997;277(4):307-11.

6. Gandhi TK, Burstin HR, Cook EF, Puopolo AL, Haas JS, Brennan TA, et al. Drug complications in outpatients. J Gen Intern Med. 2000;15(3):149-54.

7. Donaldson MS, Corrigan JM, Kohn LT. To err is human: building a safer health system. National Academies Press; 2000.

8. Agrawal A. Medication errors: prevention using information technology systems. Br J Clin Pharmacol. 2009;67(6):681-6.

9. Zhou S, Kang H, Gong Y. Design a Learning-Oriented Fall Event Reporting System Based on Kirkpatrick Model. Stud Health Technol Inform. 2017;245:828-32.

10. Macrae C. The problem with incident reporting. BMJ Qual Saf. 2016;25(2):71-5.

11. Wang Y, Coiera E, Runciman W, Magrabi F. Using multiclass classification to automate the identification of patient safety incident reports by type and severity. BMC Medical Informatics and Decision Making. 2017;17(1):84.

12. Zhou S, Kang H, Yao B, Gong Y. Unveiling Originated Stages of Medication Errors: An Automated Pipeline Approach. Stud Health Technol Inform. 2018;250:182-6.

13. Tafti A, Badger J, LaRose E, Shirzadi E, Mahnke A, Mayer J, et al. Adverse Drug Event Discovery Using Biomedical Literature: A Big Data Neural Network Adventure. JMIR Med Inform. 2017;5(4):e51.

14. Bian J, Topaloglu U, Yu F. Towards Large-scale Twitter Mining for Drug-related Adverse Events. Shb12 (2012). 2012;2012:25-32.

15. Sarker A, Gonzalez G. Portable automatic text classification for adverse drug reaction detection via multi-corpus training. Journal of biomedical informatics. 2015;53:196-207.

16. Yang M, Kiang M, Shang W. Filtering big data from social media–Building an early warning system for adverse drug reactions. Journal of biomedical informatics. 2015;54:230-40.

17. Rastegar-Mojarad M, Elayavilli RK, Wang L, Prasad R, Liu H, editors. Prioritizing adverse drug reaction and drug repositioning candidates generated by literature-based discovery. Proceedings of the 7th ACM International Conference on Bioinformatics, Computational Biology, and Health Informatics; 2016: ACM.

18. Harpaz R, DuMouchel W, Shah NH, Madigan D, Ryan P, Friedman C. Novel data‐mining methodologies for adverse drug event discovery and analysis. Clinical Pharmacology & Therapeutics. 2012;91(6):1010-21.

19. Iyer SV, Harpaz R, LePendu P, Bauer-Mehren A, Shah NH. Mining clinical text for signals of adverse drug-drug interactions. Journal of the American Medical Informatics Association. 2013;21(2):353-62.

20. Wang Y, Coiera E, Runciman W, Magrabi F. Using multiclass classification to automate the identification of patient safety incident reports by type and severity. BMC Med Inform Decis Mak. 2017;17.

21. Kang H, Wang F, Zhou S, Miao Q, Gong Y. Identifying and Synchronizing Health Information Technology (HIT) Events from FDA Medical Device Reports. Stud Health Technol Inform. 2017;245:1048-52.

22. Liang C, Gong Y. Automated Classification of Multi-Labeled Patient Safety Reports: A Shift from Quantity to Quality Measure. Studies in health technology and informatics. 2017;245:1070-4.

23. Liang C, Gong Y. Predicting Harm Scores from Patient Safety Event Reports. Studies in health technology and informatics. 2017;245:1075-9.

24. World Health Organization. Medication Errors Technical Series on Safer Primary Care [Internet]. 2016 [cited 16 July 2018]. Available from: <http://apps.who.int/iris/bitstream/10665/252274/1/9789241511643-eng.pdf>.

25. FDA. Medication Error Reports [Internet]. 2017 [cited 6 July 2018]. Available from: <https://www.fda.gov/Drugs/DrugSafety/MedicationErrors/ucm080629.htm>.

26. AHRQ. Medication Errors [Internet]. 2017 [cited 6 July 2018]. Available from: <https://psnet.ahrq.gov/primers/primer/23/medication-errors>).

27. NCC MERP. NCC MERP Taxonomy of Medication Errors [Internet]. 2001 [cited 13 July 2018]. Available from: <https://www.nccmerp.org/sites/default/files/taxonomy2001-07-31.pdf>.

28. Santell JP, Hicks RW, McMeekin J, Cousins DD. Medication errors: experience of the United States Pharmacopeia (USP) MEDMARX reporting system. J Clin Pharmacol. 2003;43(7):760-7.

29. Forrey RA, Pedersen CA, Schneider PJ. Interrater agreement with a standard scheme for classifying medication errors. Am J Health Syst Pharm. 2007;64(2):175-81.

30. Harispe S, Ranwez S, Janaqi S, Montmain J. Semantic similarity from natural language and ontology analysis. Synthesis Lectures on Human Language Technologies. 2015;8(1):1-254.

31. Pesquita C, Faria D, Falcão AO, Lord P, Couto FM. Semantic Similarity in Biomedical Ontologies. PLoS Comput Biol. 2009;5(7).

32. Pekar V, Staab S, editors. Taxonomy learning: factoring the structure of a taxonomy into a semantic classification decision. Proceedings of the 19th international conference on Computational linguistics-Volume 1; 2002: Association for Computational Linguistics.

33. Resnik P. Using information content to evaluate semantic similarity in a taxonomy. arXiv preprint cmp-lg/9511007. 1995.

34. He H, Lin J, editors. Pairwise word interaction modeling with deep neural networks for semantic similarity measurement. Proceedings of the 2016 Conference of the North American Chapter of the Association for Computational Linguistics: Human Language Technologies; 2016.

35. Benabderrahmane S, Smail-Tabbone M, Poch O, Napoli A, Devignes M-D. IntelliGO: a new vector-based semantic similarity measure including annotation origin. BMC bioinformatics. 2010;11(1):588.

36. Chabalier J, Mosser J, Burgun A. A transversal approach to predict gene product networks from ontology-based similarity. BMC bioinformatics. 2007;8(1):235.

37. Kang H, Gong Y. Developing a similarity searching module for patient safety event reporting system using semantic similarity measures. BMC Med Inform Decis Mak. 2017;17(Suppl 2):75.

38. AHRQ. Common Formats for Event Reporting - Hospital Version 2.0: Agency for Healthcare Research and Quality [Internet]. 2017 [cited 15 July 2018]. Available from: <https://www.psoppc.org/psoppc_web/publicpages/commonFormatsHV2.0>.

39. Porter MF. Snowball: A language for stemming algorithms. 2001.

40. McCallum A. Rainbow [Internet]. 1998 [cited 10 July 2018]. Available from: <http://www>.cs.cmu.edu/mccallumbow.

41. Sivic J, Zisserman A. Efficient visual search of videos cast as text retrieval. IEEE transactions on pattern analysis and machine intelligence. 2009;31(4):591-606.

42. Salton G, Buckley C. Term-weighting approaches in automatic text retrieval. Information processing & management. 1988;24(5):513-23.

43. Lee C, Lee GG. Information gain and divergence-based feature selection for machine learning-based text categorization. Information processing & management. 2006;42(1):155-65.

44. Ng AY, Jordan MI, editors. On discriminative vs. generative classifiers: A comparison of logistic regression and naive bayes. Advances in neural information processing systems; 2002.

45. Hsu C, Chang C, Lin C. A practical guide to support vector classification [Internet]. 2003 [cited 15 July 2018]. Available from: https://www.csie.ntu.edu.tw/~cjlin/papers/guide/guide.pdf

46. Huang A, editor Similarity measures for text document clustering. Proceedings of the sixth new zealand computer science research student conference (NZCSRSC2008), Christchurch, New Zealand; 2008.

47. Institute for Healthcare Improvement. How-to Guide: Prevent Harm from High-Alert Medications [Internet]. 2012 [cited 16 July 2018]. Available from: <http://www.ihi.org/resources/Pages/ToolsHowtoGuidePreventHarmfromHighAlertMedications.aspx>.

**Table 1**

Labels in event originated stage, event type and event cause.

| Attributes | Labels per NCC MERP Taxonomy |
| --- | --- |
| Event Originated Stage | “ordering”, “transcribing”, “dispensing”, “administering”, “monitoring”, “medication reconciliation” |
| Event Type | “wrong dose”, “wrong dose (omission)”, “wrong drug”, “wrong time”, “wrong record”, “billing issue”, “adverse drug reaction” and “wrong administration” |
| Event Cause | “information deficit”, “performance deficit”, “devices (HIT)”, “pathophysiological factor”, and “external factor” |

**Table 2**

An example of similar medication event reports

| **Cases** | **Report Details** |
| --- | --- |
| Report_1 | Patient ordered: Take 1/2 of Drug A 0.5mg tab for total dosage of 0.25mg TID. When looking at the narc book to check what had been signed out since yesterday I noticed that [x] who gave the patient's AM dose did not 1/2 the tablet that she gave. I double checked with the destruction log to see if anything was wasted and it was not. Patient received 0.5mg instead of 0.25mg. Informed adult day nurse [x] who will follow up with the charge nurse and inform the physician. |
| Report_2 | 39 units of Drug A drawn and administered instead of the required 14 units of Drug A as ordered. (Does have an order for 25 units of Drug A not QID) |

**Table 3**

An example of designed multiple-choice question in questionnaire.

| **Reports** | **Report Details** |
| --- | --- |
| **Target Report** | Patient given 60 mg Drug A ivp 4.5 hours early than scheduled time. Dr. [x] called and said hold Drug B for two hours. Pt showing no signs or symptoms of reaction to early dose. |
| **A** | Patient was ordered Drug A 0.1mg PO QHS. The order was put in with the correct directions and wrong time. [x] gave the patient Drug A 0.1mg at 06:30 instead of 21:30 on 9/16/16. Pharmacy did not merge the manual order yesterday with their order so the patient also received 0.1mg at 21:30 on 9/15/16. I discontinued the manual order and informed [x]. |
| **B** | Patient given Drug A and developed redness and rash, drug discontinued, given Drug B. |
| **C** | Pyxis drawer failed and never opened when trying to remove 4 5mg Drug A. Drawer then recovered with [x]. oxy count was then off, report showed that I had pulled the meds which I had not. [x] was also a witness. |
| **D** | I went into the room at 16:30, to give the patient her 17:00 meds. While in the room, I asked the patient if she was in pain. She stated she was and would like a pain pill. Without double checking the MAR I pulled the patients Drug A and gave it to her. When I informed the nurse that I had given her the drug, she stated the next dose is scheduled at 20:00. |

**Table 4**

Performances of ZeroR classifier for identifying the error originated stages, types and causes.

| **Classification Task** | **Overall Precision** | **Overall Recall** | **Overall F-Measure** |
| --- | --- | --- | --- |
| Event Originated Stage | 0.234 | 0.484 | 0.315 |
| Event Type | 0.139 | 0.373 | 0.203 |
| Event Cause | 0.256 | 0.506 | 0.340 |

**Table 5**

SVM Implementation for Identifying the Event Originated Stages

| **Event Originated Stage** | **Precision** | **Recall** | **F-Measure** |
| --- | --- | --- | --- |
| Ordering | 0.895 | 0.892 | 0.894 |
| Transcribing | 0.464 | 0.430 | 0.446 |
| Dispensing | 0.612 | 0.502 | 0.552 |
| Administering | 0.735 | 0.797 | 0.765 |
| Monitoring | 0.768 | 0.730 | 0.748 |
| Medication Reconciliation | 0.778 | 0.700 | 0.737 |
| Overall | 0.792 | 0.795 | 0.792 |

**Table 6**

SVM Implementation for Identifying the Event Types

| **Eevnt Type** | **Precision** | **Recall** | **F-Measure** |
| --- | --- | --- | --- |
| Adverse Drug Reaction | 0.766 | 0.873 | 0.816 |
| Billing Issues | 0.978 | 0.978 | 0.978 |
| Wrong Dose | 0.493 | 0.540 | 0.516 |
| Wrong Dose (Omission) | 0.640 | 0.550 | 0.591 |
| Wrong Record | 0.871 | 0.857 | 0.864 |
| Wrong Drug | 0.497 | 0.682 | 0.575 |
| Wrong Time | 0.621 | 0.143 | 0.232 |
| Wrong Administration | 0.727 | 0.129 | 0.219 |
| Overall | 0.778 | 0.769 | 0.758 |

**Table 7**

Random Forest Implementation for Identifying the Event Causes

| **Event Cause** | **Precision** | **Recall** | **F-Measure** |
| --- | --- | --- | --- |
| Performance Deficit | 0.856 | 0.978 | 0.913 |
| Information Deficit | 0.714 | 0.070 | 0.128 |
| Devices (HIT) | 0.632 | 0.126 | 0.210 |
| Pathophysiological Factor | 0.896 | 0.628 | 0.738 |
| External Factor | 0.979 | 0.947 | 0.963 |
| Overall | 0.927 | 0.927 | 0.925 |

**Table 8**

Accuracies for the 11 participants under two standards

| Participant ID | 1 | 2 | 3 | 4 | 5 | 6 | 7 | 8 | 9 | 10 | 11 |
| --- | --- | --- | --- | --- | --- | --- | --- | --- | --- | --- | --- |
| Accuracy  (strict standard) | 90% | 70% | 90% | 20% | 80% | 100% | 100% | 90% | 100% | 60% | 90% |
| Accuracy  (loose standard) | 100% | 90% | 100% | 50% | 100% | 100% | 100% | 100% | 100% | 90% | 100% |

**Fig 1**.

Overall sketches for the proposed automated pipeline for analysis of medication event reports

**Fig 2**.

Distributions of the annotated event originated stages of the medication event reports

**Fig 3**.

Distributions of the annotated event types of the medication event reports

**Fig 4**.

Distributions of the annotated event causes of the medication event reports

**Fig 5**.

Accuracies of the each multiple-choice question in the questionnaire under two standards


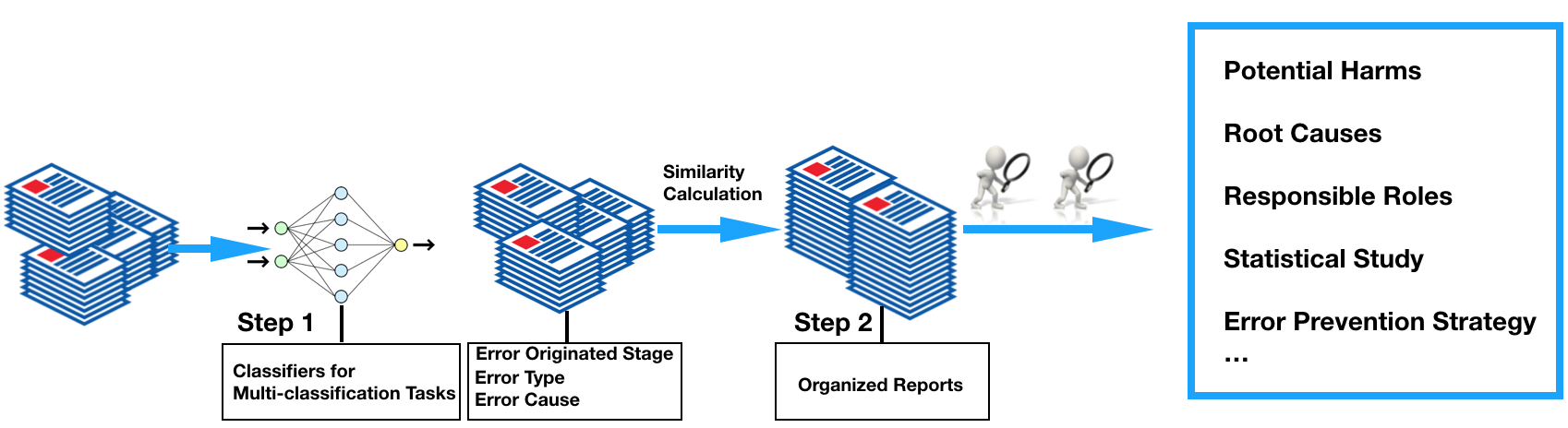


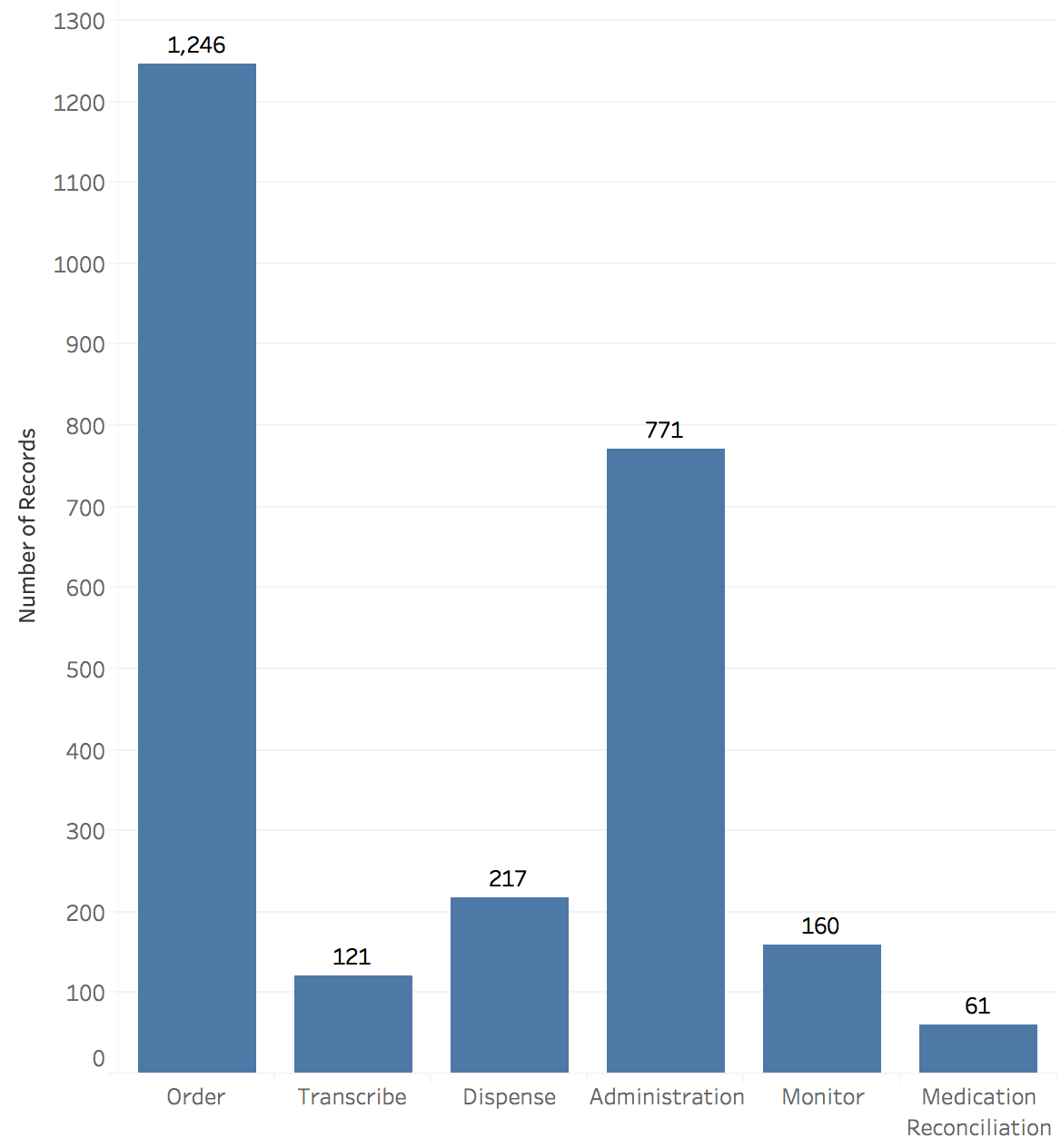


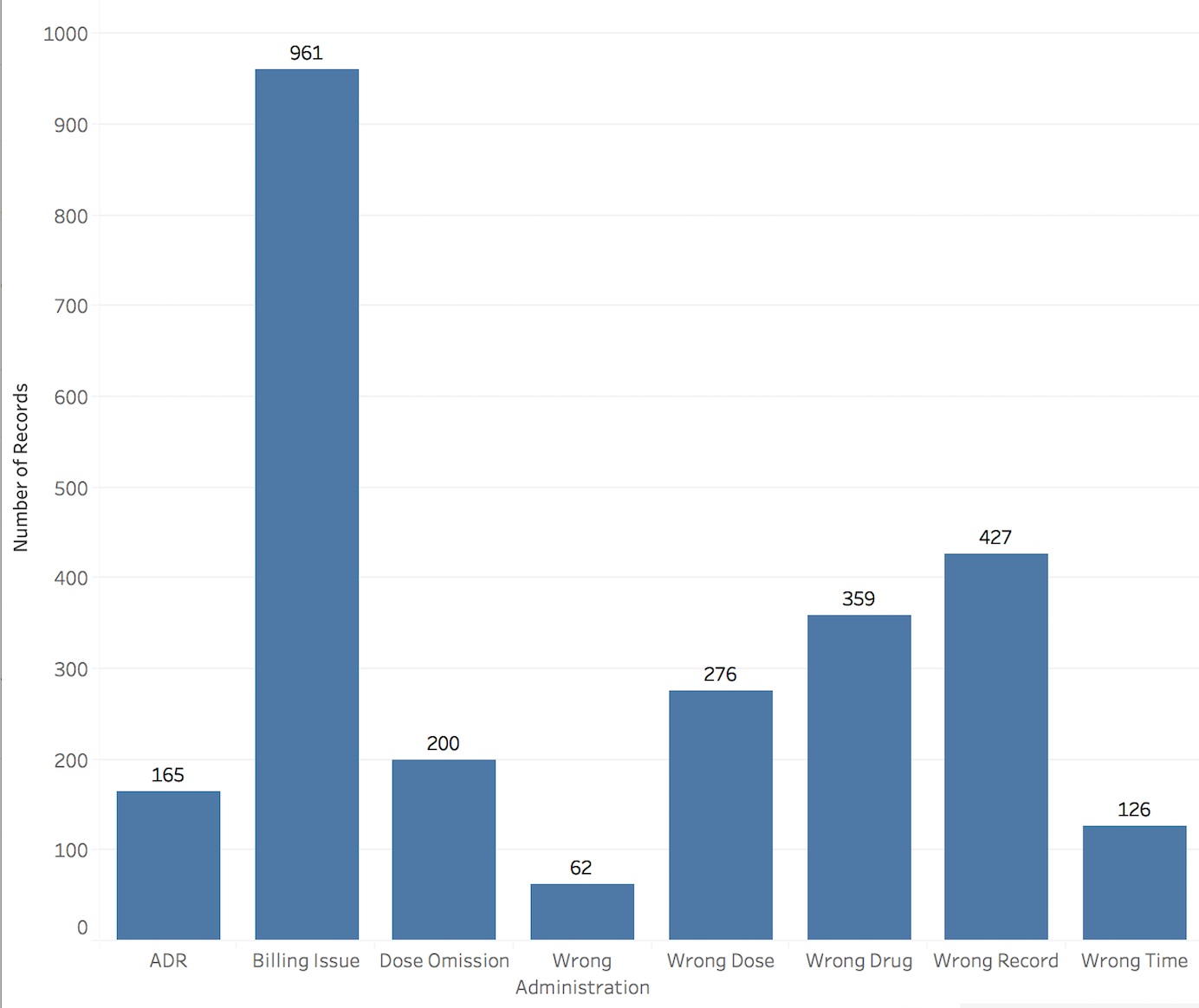


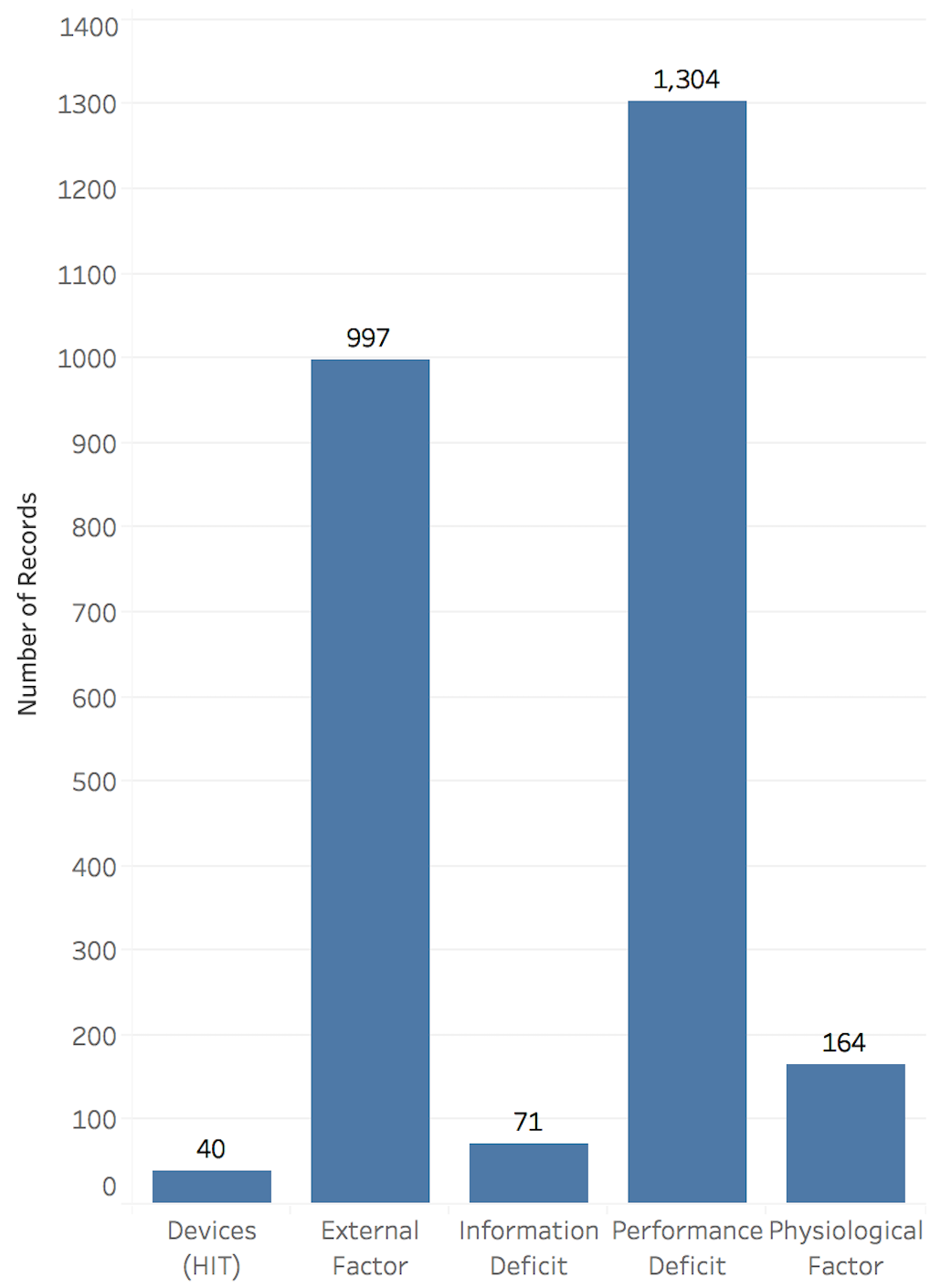


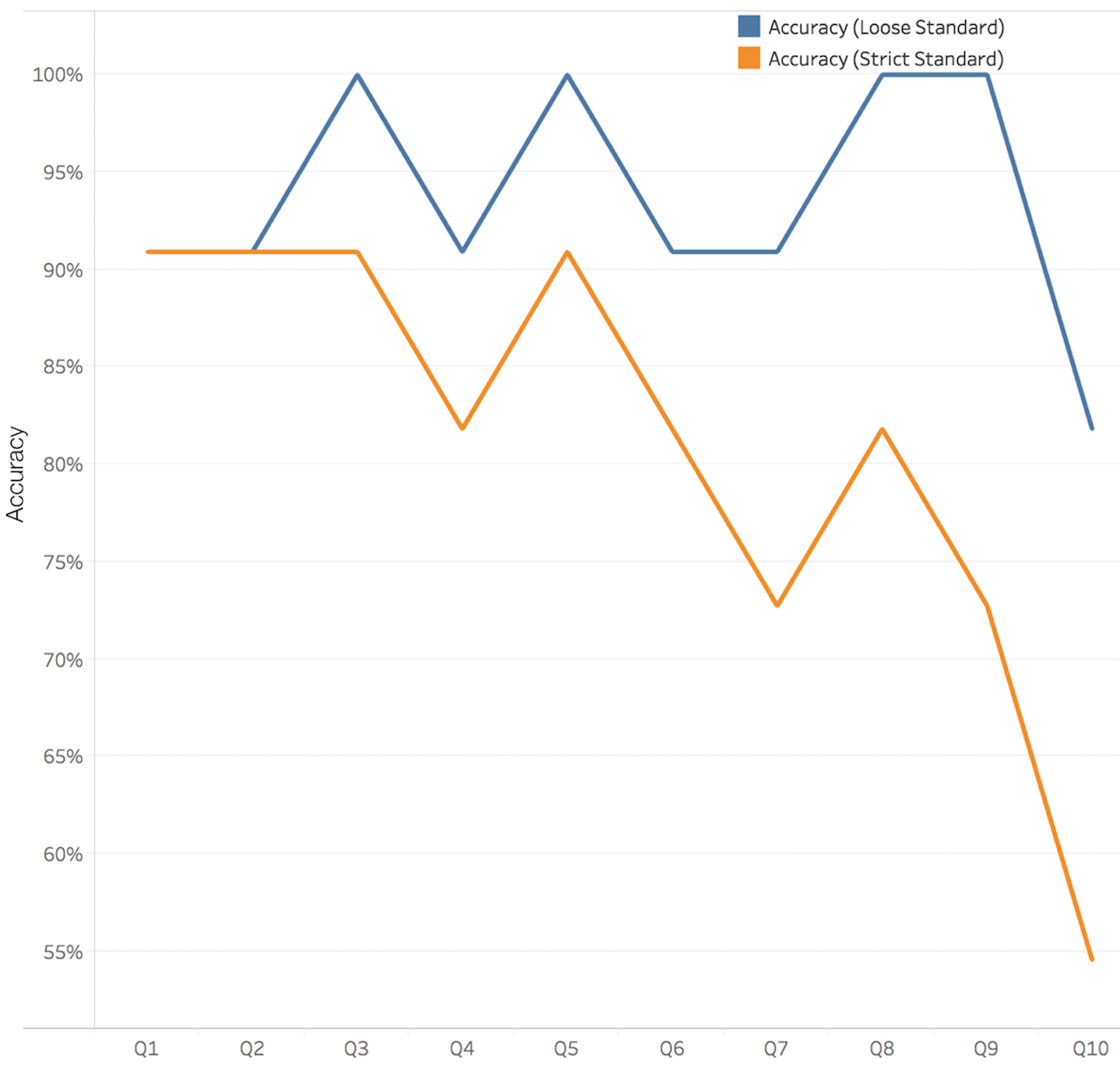

Supplement: Supplementary file 1 — An automated pipeline for analyzing medication event reports in clinical settings. (DOCX 1077 kb) [file 12911_2018_687_MOESM1_ESM.docx]
